# Case Report: Infected primary hydatid cyst of the thigh

**DOI:** 10.12688/f1000research.53651.1

**Published:** 2021-08-10

**Authors:** Myriam Jrad, Haifa Zlitni, Hakim Zouari, Miriam Boumediene, Ines Soussi, Meriem Bouzrara

**Affiliations:** 1 Department of Radiology, Hospital Charles Nicolle, Tunis, 9 April Bd, Bab Souika, Tunis, 1006, Tunisia

**Keywords:** hydatidosis, cyst, ultrasound, MRI, surgery

## Abstract

Hydatic cyst may occur in many organs such as the liver, lung, brain or heart with radiologic features of liver and lung involvement being well known. The musculo-skeletal site is infrequent accounting for 0.7–3% cases of all cases resulting from direct implantation of oncospheres more often than hematic dissemination.

We report the case of an 18-year-old female student who visited our hospital because of a swelling in the posteroexternal aspect of the left thigh that had grown during the previous six months and had become tender in the previous month with setup of fever three days before admission. Superficial ultrasound and magnetic resonance imaging demonstrated a cystic mass of the posterior compartment of the thigh developed within the short chief of the biceps femoris. Serology for hydatid cyst was positive. The diagnosis of an infected hydatid cyst was suspected preoperatively, and the patient was given antibiotics and anthelminthic treatment. The cyst was then completely excised and the histopathologic exam confirmed the hydatic origin. The patient was put on oral anti-helminthics and has been on regular follow up for last twelve months with no evidence of recurrence.

Hydatidosis rarely occurs in the soft tissues and the diagnosis is challenging particularly when it is secondary infected. Hydatid serology provides certainty in the diagnosis of echinococcosis when it is positive. When it’s negative, imaging (Ultrasound, Computed tomography (CT) and Magnetic resonance imaging (MRI)) may be an approach for making the diagnosis revealing the most characteristic features of hydatid cyst.

## Case presentation

An 18-year-old Tunisian female student presented to the orthopedics department of Charles Nicolle Hospital of Tunis, Tunisia on January 15, 2019 with a lump in the posteroexternal aspect of the left thigh. She had noticed the swelling on her thigh six months before visiting the hospital. She was without history of trauma, surgery or any additional disease. The swelling had become painless during the six months prior to her visit but it had become tender within the previous month with the setup of fever three days prior. On examination, the patient was febrile (38.5° Celsius) with normal vital parameters. There was a tender, indurate, non-moveable lump on the posteroexternal aspect of the middle one-third of the left thigh measuring about 12 cm × 5 cm. The overlying skin was erythematous without any punctum or discharge. The knee and leg movements were normal.

**Figure 1.**
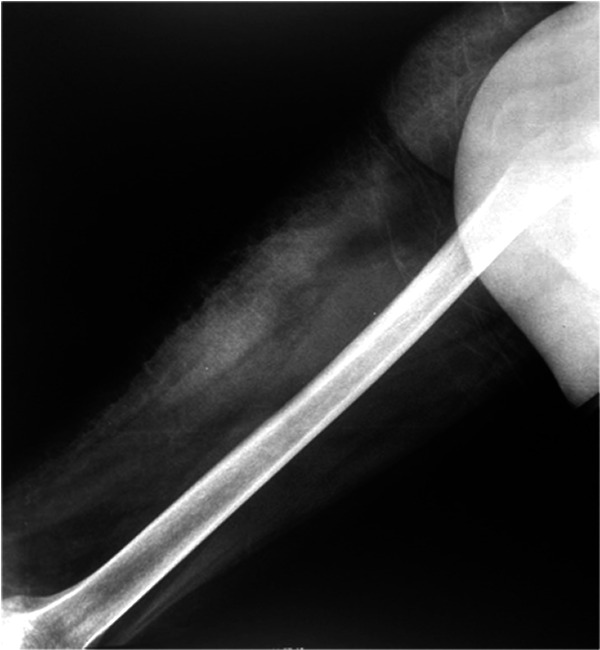
Conventional radiography of the left femur showing a thickening of the soft tissues of the middle and posterior region of the thigh with integrity of the bone.

Laboratory investigations on January 15, 2019 showed a biological inflammatory syndrome with elevated white blood cell count (12,000/mm ^3^) and C-reactive protein. Conventional radiography of the left femur showed a thickening of the soft tissues of the middle and posterior region of the thigh with integrity of the bone ( Figure 1).

**Figure 2.**
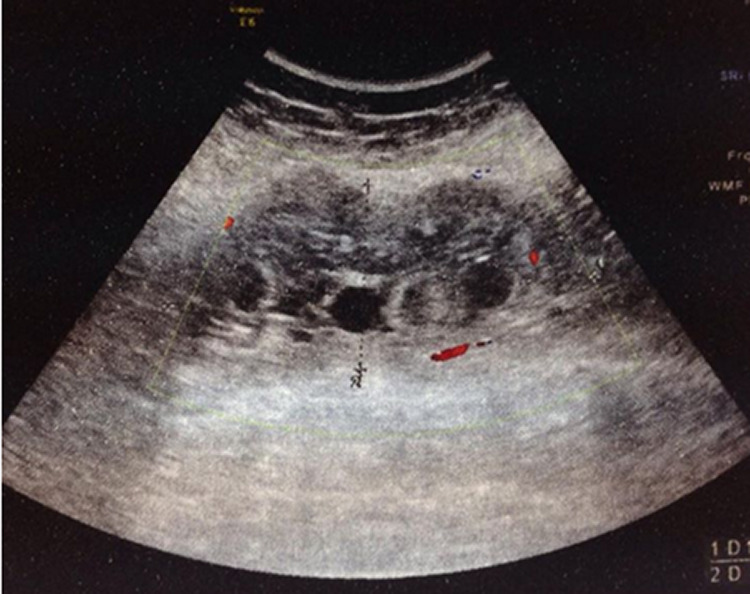
Superficial Doppler ultrasound in the transversal plane (A) and longitudinal plane (B) of the thigh shows an ill-defined multilocular cystic mass of the middle one-third of the posterior compartment of the thigh measuring 13 cm × 5.5 cm and containing an echogenic peripheral portion that is finely vascularized on color Doppler.

**Figure 3.**
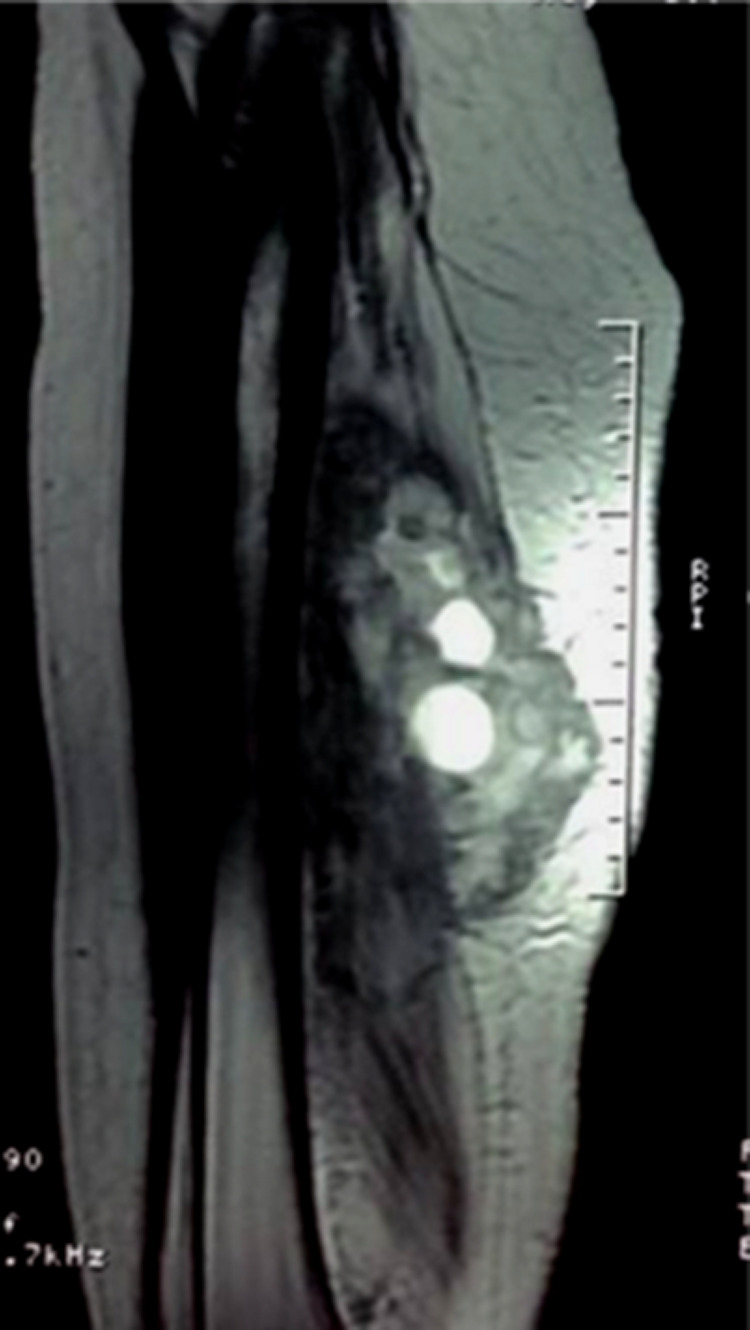
MRI of the left thigh: Sagittal TSE T2-weighted image shows the multiloculated cystic lesion with multiple daughter cysts in the middle one-third of the posterior compartment of thigh.

**Figure 4.**
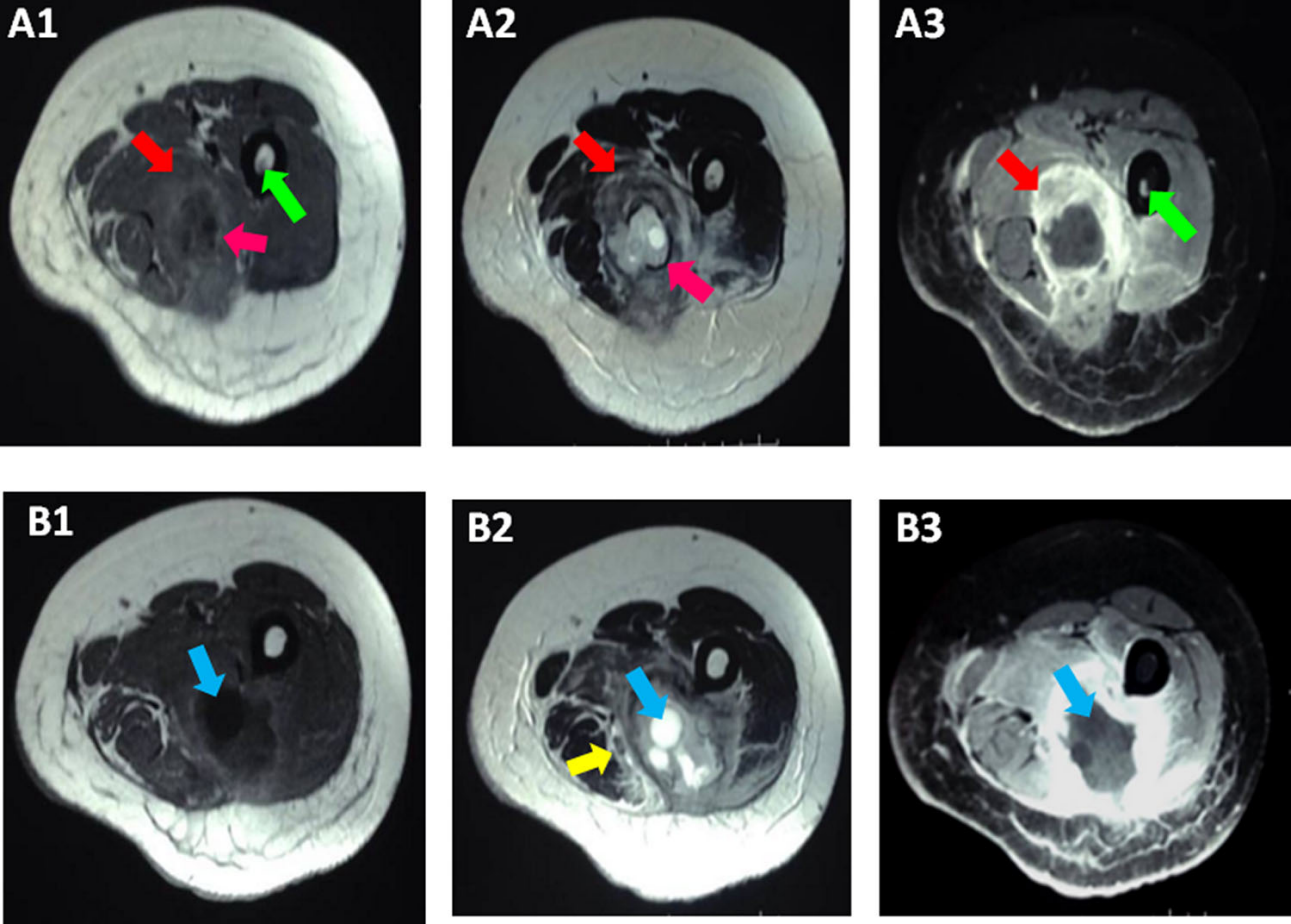
MRI of the left thigh in two different cutting levels (A1+A2+A3) and (B1+B2+B3). Axial TSE T1-weighted images (A1+B1), TSE T2-weighted images (A2+B2) and contrast-enhanced fat-suppressed TSE T1-weighted images demonstrated an intramuscular cystic mass of the middle one-third of the posterior compartment of the left thigh within the biceps femoris muscle. This mass is delimited by a discontinuous rim of low T2 and high T1 signal "rim sign"(pink arrow) and contains multiple well defined cystic lesions of more intense high T2 and low T1 signal corresponding to daughter cysts (blue arrow) with a "cyst within a cyst” appearance. This cystic mass is surrounded by an edematous infiltration of the adjacent muscles with low T1 and high T2 signal and avid enhancement after contrast administration (red arrows). Enhancement of the muscular fascia and of the subcutaneous fat of the posterior aspect of thigh. The mass represses the sciatica nerve without invading it (yellow arrow) and is distant from the profound and superficial femoral pedicles. A low T1 signal of the spongiest bone enhanced after contrast administration was noted (green arrow).

Superficial Doppler ultrasound performed the second day of hospitalization showed an ill-defined multilocular cystic mass of the middle one-third of the posterior compartment of the left thigh measuring 13 cm × 5.5 cm and containing an echogenic peripheral portion that was finely vascularized on color Doppler ( Figure 2). Magnetic resonance imaging (MRI) performed two days later demonstrated a large intramuscular cystic mass of the middle one-third of the posterior compartment of the left thigh ( Figure 3) within the biceps femoris muscle measuring 10 cm × 4 cm. This mass was delimited by a discontinuous rim of low T2 and high T1 signal “rim sign” and contained multiple well defined cystic lesions of more intense high T2 and low T1 signal corresponding to daughter cysts with a “cyst within a cyst appearance”. This cystic mass was surrounded by an edematous infiltration of the adjacent muscles with low T1 and high T2 signal and avid enhancement after contrast administration predominant in the posterior compartment. Enhancement of the muscular fascia and of the subcutaneous fat of the posterior aspect of the thigh was noticed. The mass repressed the sciatica nerve without invading it and was distant from the profound and superficial femoral pedicles. A low T1 signal of the spongy bone enhanced after contrast administration was noticed ( Figure 4).

The enzyme-linked immune-absorbent assay (ELISA) was positive for the *Echinococcal granulosis* antigens (40 U/ml).

The diagnosis of an infected hydatid cyst was suspected perioperatively and the patient was given antibiotics and anthelminthic treatment (Albendazole 400 mg Per Os twice daily for 28 days). The patient didn't have any history of hydatidosis and hydatid cysts were not detected in any other organ on preoperative computed tomography (CT) of the abdomen and thorax. The surgical exploration found a firm oblong mass within the short chief of left biceps femoris densely adherent to the surrounding muscles and abutting the femur cortex. The mass was widely excised. The surgeon then performed an irrigation with Povidone iodine and hypertonic saline solutions and closed the wound over a negative suction drain. The macroscopic examination of the lesion revealed multiple daughter cysts and the histopathological exam confirmed the hydatic origin.

## Discussion

Echinococcosis is a cosmopolitan helminthic infection caused by the tapeworm *Echinococcus granulosus* and it affects humans and many mammals. ^1^ This tapeworm species is endemic in the Mediterranean region, Australia, Argentina, Africa, Eastern Europe and the Middle East. The dog is a definitive host, but this situation is shared by the wolf and some species of jackal. ^2^

The dog infestation is through the digestive track and is believed to be secondary to the consumption of parasite viscera especially the liver and the lungs of the sheep as an intermediate host. ^3^ The latter, constituting the main reservoir of *Echinococcus* tapeworm, becomes infected by eating grass soiled by the dog’s droppings containing the eggs of the parasite. ^4^ Humans are only an intermediate host and an epidemiological impasse of the parasite. They become infected either through direct contact with parasitized dogs or indirectly through ingestion of contaminated food. ^3– 5^

Muscular localization of hydatid cyst is rare varying from 1 to 5.4 % of all hydatid locations. ^6^ It’s the third localization after the lungs and the liver. For some, involvement of the spleen must precede that of muscle since it is estimated at 8%. ^1, 7^ Several arguments have been put forward to explain the scarcity of muscle localization: the efficiency of hepatic and pulmonary barriers that opposes the migration of the parasite into the systemic circulation; the muscular environment’s hostility to the growth of hydatid larvae due to the production of lactic acid and the alternation of contraction–relaxation inhibiting uniform vascularization. ^8, 9^ The muscle localization of echinococcosis seems to be mostly primary and affects mainly proximal muscles of the lower limbs, very probably due to the importance of irrigation of these. ^10^ Daali and Hssaida reported 10 cases out of 15 of deep muscular location involving the diaphragm and psoas. ^11^

Diagnosis of echinococcosis must be suspected when a patient from a rural area is presenting with slowly growing soft tissue mass and it should be included in the differential diagnosis of limb masses: abscess, malignant or benign tumor, calcified hematoma or lipoma. ^12^ The diagnosis of echinococcosis should be considered before surgical biopsy in order to prevent the risk of anaphylaxis. ^13^

Ultrasound is a non-irradiating, accessible, and non-expensive exam, which can be used as a first line approach for making the diagnosis revealing the most characteristic features of hydatid cyst: daughter cysts, detached membranes and double line sign. Cysts may be classified according to the ultrasound criteria of Gharbi. ^14^ Atalar *et al.* reported a sensitivity of 95%, increasing to 100% in the presence of vesicular fibrils. ^15^ In our case, superficial Doppler ultrasound showed a multilocular cystic mass (type III) of the middle one-third of the posterior compartment of the left thigh. The mass was ill-defined and containing an echogenic peripheral portion finely vascularized on color Doppler probably related to the secondary infection of the cyst. However, in non-endemic areas, ultrasound can be misleading with soft tissue tumors especially in deep locations. ^16^

Computed tomography (CT) radiologic features vary from a unilocular or multilocular cyst, with or without septas, debris or wall calcifications, to a complex or solid mass without enhancement on intravenous contrast. ^13^ The appearance of muscular hydatidosis is unoften typical and the multivesicular form is specific as is reflects multiple daughter cysts within the parent cyst. Since bony invasion and relationship of the cyst with adjacent organs is essential to describe, computed tomography (CT) must be a part of the screening protocol. ^17^

Although, magnetic resonance imaging (MRI) characteristics of liver hydatid cyst are detailed in the literature, the diagnosis is challenging in the soft tissue of the musculoskeletal system because the magnetic resonance imaging (MRI) features are not well labelled. Magnetic resonance imaging (MRI) is the gold standard imaging test in the identification of soft-tissue masses including hydatid disease thanks to its capacity to establish most of its features, with the exception of calcifications. Performing magnetic resonance imaging (MRI) requires the use of a surface antenna depending on the concerned part of the body, the use of a large field of view allowing the inclusion of the neighboring joint, a section thickness of 3 to 7 mm, and an inter-cut space of 0 to 2 mm. Acquisitions are performed in the axial plan, sagittal plan for anterior or posterior lesions, and coronal plan for lateral or medial lesions. The sequences must include T1-weighted sequence in the axial plane and T2 and T1-weighted sequences after fat saturation before and after injection of gadolinium in two orthogonal planes.

The classic magnetic resonance imaging (MRI) findings include a unilocular or multilocular cyst with a low-intensity rim ("rim sign") or detached membrane on T2-weighted images without enhancement after contrast injection. ^18^ “The rim sign” corresponds to the pericyst that is a collagen reaction generated by the host. The most pathognomonic sign is that of daughter cysts within a larger cyst. ^19^ The rim sign is a characteristic sign in muscular hydatidosis that is uncommon in hydatic cyst located elsewhere in the body. Magnetic resonance imaging (MRI) of our patient demonstrated a cystic mass containing multiple well-defined cysts corresponding to daughter cysts with a “cyst within a cyst appearance” delimited by a discontinuous “rim sign”. ^20^ This lesion was surrounded by an edematous infiltration with avid enhancement of the muscular environment and the spongy bone in contact after contrast related to the secondary infection of the cyst. ^19– 21^

Hydatid serology provides certainty in the diagnosis of echinococcosis when it is positive. However, there is a significant proportion of false negatives, variable depending on the location of the cyst. Lamine *et al.* reported 80% of false negatives. ^9^ The enzyme-linked immune-absorbent assay (ELISA) was positive for the *E. granulosis* antigens in our case. Hypereosinophilia is not specific and inconstant and is of interest only in the orientation of the diagnosis, ultrasound, and nowadays magnetic resonance imaging (MRI) can confirm the diagnosis. ^9, 19^

Surgical excision of the cyst is the treatment of choice. For non-surgical cysts, anthelminthic chemotherapy with or without percutaneous aspiration-injection re-aspiration (PAIR) is an alternative option for the treatment. ^21, 22^ Our patient was given antibiotics and anthelminthic treatment (Albendazole 400 mg Per Os twice daily for 28 days). Thereafter, the mass was widely excised, and the patient was put on oral anthelminthics after surgery. Percutaneous drainage echo guided without re-aspiration is simple, easy to apply, low cost, repeatable, and does not require hospitalization. ^21, 22^

Hydatidosis rarely occurs in the soft tissues and the diagnosis is challenging particularly when it is secondary infected. Hydatid serology provides certainty in the diagnosis of echinococcosis when it is positive. When it’s negative, ultrasound is an accessible way to approach the diagnosis, computed tomography (CT) may help to evaluate the surrounding tissues and find calcifications and magnetic resonance imaging (MRI) provides imaging characteristics of hydatic cyst. Open surgery is the gold standard of the treatment of muscular hydatidosis while ambulatory percutaneous techniques are gaining scale.

## Data availability

All data underlying the results are available as part of the article and no additional source data are required.

## Consent

Written informed consent for publication of clinical details and clinical images was obtained from the patient.
