# Supplementary material for: Case Report: Infected primary hydatid cyst of the thigh
Source: F1000Res. 2021 Aug 10;10:786. [Version 1] doi: 10.12688/f1000research.53651.1 (PMC11923533; doi:10.12688/f1000research.53651.1)
Supplement: Peer review history

# Reviewer report: Reviewer response for version 1

**DOI:** 10.5256/f1000research.57064.r368921

**Published:** 2025-03-19

**Authors:** Rahul Gupta

**Affiliations:** 1 Synergy Institute of Medical Sciences, Dehradun, India

The authors have presented a case of infected hydatid cyst. Please clarify the following details:

 1. How long was albendazole given preoperatively?

 2. How to differentiate musculoskeletal hydatid cyst from cysticercosis on radiology?

 3. Please add postoperative follow up of the patient in the Case presentation.

 4. The discussion section is too lengthy and must discuss the differential diagnoses.

Are enough details provided of any physical examination and diagnostic tests, treatment given and outcomes?

Yes

Is the case presented with sufficient detail to be useful for other practitioners?

Yes

Is sufficient discussion included of the importance of the findings and their relevance to future understanding of disease processes, diagnosis or treatment?

Yes

Is the background of the case’s history and progression described in sufficient detail?

Yes

Reviewer Expertise:

General surgery

I confirm that I have read this submission and believe that I have an appropriate level of expertise to confirm that it is of an acceptable scientific standard.

## References

[1] : The unsolved problem of musculoskeletal hydatid disease: two case reports. *J Med Case Rep* .2023;17(1) : 10.1186/s13256-023-04275-4 531 10.1186/s13256-023-04275-4 38148487 PMC10752011

# Reviewer report: Reviewer response for version 1

**DOI:** 10.5256/f1000research.57064.r102033

**Published:** 2021-12-23

**Authors:** Hichem Jerraya

**Affiliations:** 1 Department B of General Surgery, Faculty of Medicine of Tunis, Charles Nicolle Hospital, Tunis El Manar University, Tunis, Tunisia

I congratulate the authors for the quality of their manuscript. The iconography is rich and of high value.

 I have only two comments to make:

1. In the abstract, one has the impression that hydatid serology represents an indispensable tool in the diagnostic process. I think that currently in endemic countries, serology has been supplemented by imaging and its only interest could be in rare cases of diagnostic doubt after complete morphological assessment.
2. Once the diagnosis of infected hydatid cyst has been retained, should the surgery have been indicated earlier? and was intraoperative samples done to confirm the diagnosis of hydatid cyst infection?

Are enough details provided of any physical examination and diagnostic tests, treatment given and outcomes?

Yes

Is the case presented with sufficient detail to be useful for other practitioners?

Yes

Is sufficient discussion included of the importance of the findings and their relevance to future understanding of disease processes, diagnosis or treatment?

Yes

Is the background of the case’s history and progression described in sufficient detail?

Yes

Reviewer Expertise:

hydatid cyst surgery; digestive surgery

I confirm that I have read this submission and believe that I have an appropriate level of expertise to confirm that it is of an acceptable scientific standard.

## References

[1] : Echinococcosis: Advances in the 21st Century. *Clinical Microbiology Reviews* .2019;32(2) : 10.1128/CMR.00075-18 10.1128/CMR.00075-18 PMC643112730760475
